# Early cardiac toxicity following adjuvant radiotherapy of left-sided breast cancer with or without concurrent trastuzumab

**DOI:** 10.18632/oncotarget.6053

**Published:** 2015-10-09

**Authors:** Lu Cao, Gang Cai, Cai Chang, Zhao-Zhi Yang, Yan Feng, Xiao-Li Yu, Jin-Li Ma, Jiong Wu, Xiao-Mao Guo, Jia-Yi Chen

**Affiliations:** ^1^ Department of Radiation Oncology, Fudan University Shanghai Cancer Center, Shanghai, China; ^2^ Department of Ultrasound, Fudan University Shanghai Cancer Center, Shanghai, China; ^3^ Department of Breast Surgery, Fudan University Shanghai Cancer Center, Shanghai, China; ^4^ Department of Radiation Oncology, Ruijin Hospital, Shanghai Jiaotong University School of Medicine, Shanghai, China; ^5^ Shanghai Medical College, Fudan University, Shanghai, China

**Keywords:** breast cancer, cardiotoxicity, radiotherapy, trastuzumab, concurrent treatment

## Abstract

**Purpose:**

To evaluate the influence of concurrent trastuzumab on the cardiotoxicity in patients receiving left-sided adjuvant radiotherapy.

**Materials and Methods:**

Medical records of stage I-III left-sided breast cancer patients, including 64 receiving concurrent trastuzumab with radiotherapy and 73 receiving radiotherapy alone were retrospectively reviewed. All of the patients had normal LVEF after adjuvant chemotherapy. Information of doses volume to cardiac structures was collected. Cardiac events were assessed according to CTC 2.0.

**Results:**

Median follow-up of LVEF and clinical assessment of cardiac function from the initiation of radiotherapy was 6.7 months (range 3–60.9) and 26 months (range 6.4–60.9), respectively. Grade 1 LVEF dysfunction occurred in 5 (7.8%) and 3 (4.1%) patients of the concurrent-trastuzumab and radiotherapy alone cohort, respectively. Trastuzumab was the only significant factor influencing absolute LVEF decrease in univariate analysis. In multivariate analysis of concurrent-trastuzumab cohort, IMC radiotherapy and start trastuzumab during radiotherapy were independent risk factors. For concurrent cohort, mean heart dose, as well as D_10_-D_30_, D_50_-D_55_, V_5_-V_20_ of the heart and D_30_-D_45_, D_65_-D_75_, V_6_-V_15_ of the LV were significantly higher in patients developing LVEF dysfunction.

**Conclusions:**

Concurrent trastuzumab and left-sided radiotherapy is well tolerated in terms of cardiotoxicity in patients with normal baseline cardiac function after adjuvant chemotherapy. However, increases in mean dose and low–dose volume of cardiac structures are associated with a higher risk of acute LVEF dysfunction.

## INTRODUCTION

Radiation therapy (RT) has been established to be an important treatment strategy after breast conservative surgery or after mastectomy in node positive patients [1-3]. One of the major toxicities of RT is cardiotoxicity, resulting in an increase of non-breast cancer related mortality [3-5]. Modern techniques have allowed much better protection of the heart [6-9]. However, as heart lies just below the chest wall, the risk of radiation-induced cardiac damage persists, especially in patients with left-sided breast cancer.

HER2 is expressed or amplified in about 25% of breast cancers, and is associated with increased risk for recurrence and mortality [10-12]. Trastuzumab (Herceptin; Roche, Basel, Switzerland), a HER2-directed humanized monoclonal antibody, has been shown to improve both disease-free and overall survival in HER2-positive (HER2+) breast cancer patients [13]. The most common toxicity reported from trastuzumab is cardiotoxicity [11], which presents most frequently in the form of asymptomatic decrease in left ventricular ejection fraction (LVEF) [14].

Benefit of trastuzumab is higher when administrated as soon as possible and concurrently with adjuvant chemotherapy [15, 16]. Therefore, adjuvant RT is often delivered concurrently with adjuvant trastuzumab. RT associated cardiotoxicity has been reported as a result of damage to micro- and macro-vascular structures [17, 18]. There are much fewer reports about mechanisms of trastuzumab-induced cardiotoxicity. In animal studies, it was found to cause dilated cardiomyopathy through inhibition of HER2 receptor [19]. In most cases, LVEF recovers to normal but not always baseline level after trastuzumab is discontinued without cardiologic treatment [20, 21]. It is possible that heart exposed to trastuzumab is more susceptible to the damage from ionizing irradiation.

Radiation-related cardiotoxicity depends mainly on the dose volumes of the heart irradiated [22]. In a previous retrospective study, our group found a positive association between the dose volumes histograms of cardiac structures and early cardiotoxicity in left-sided breast cancer patients receiving concurrent trastuzumab and RT. [23]. However, the limited sample size (enrolling only 24 left-sided breast cancer patients) has limited us from extrapolating the dose-volume recommendation.

The main objective of this study was first, to evaluate the influence of concurrent trastuzumab on the cardiotoxicity in patients receiving left-sided adjuvant RT. Second, to explore the detailed dosimetric information with regard to the acute systolic dysfunction, so as to provide information for future practical recommendations of dose-volume constraints in that specific clinical scenario.

## RESULTS

### Baseline characteristics

In total, 64 patients treated with concurrent trastuzumab and left-sided RT (the concurrent-trastuzumab cohort) and 73 patients treated with left-sided RT alone (the no-trastuzumab cohort) were enrolled in this analysis. Clinical features of the 137 patients according to trastuzumab treatment are shown in Table 1. The two cohorts were similar in age, BMI, comorbidity, menopausal status and clinical-pathologic stage. No patient was active smoker. All patients who received concurrent trastuzumab had HER2+ invasive ductal carcinoma. Compared with those receiving no trastuzumab, the proportion of hormone receptor (HR) negativity was higher in patients who received concurrent trastuzumab (*P* = 0.01).

**Table 1:** Baseline patient demographics and clinical characteristics

Characteristics	Concurrent trastuzumab		No trastuzumab		*P* value
	N (64)	%	N (73)	%	
Age (y), median (range)	45 (26-71)		48 (25-71)		0.537
BMI (kg/m^2^), median (range)	22.1 (16.7-28.3)		22.5 (16.6-30.5)		0.655
Comorbidity					
HD/HTN/DM	10	15.6	13	17.8	0.733
None	54	84.4	60	82.2	
Menopausal status					
Pre/peri-menopausal	49	76.6	50	68.5	0.293
Post-menopausal	15	23.4	23	31.5	
Histology type					
Ductal carcinoma	64	100	62	84.9	0.005
Lobular carcinoma	0	0	3	4.1	
Others	0	0	8	11	
Tumor stage					
T1	30	46.9	37	50.7	0.623
T2	31	48.4	28	38.4	
T3	3	4.7	4	5.5	
T4	0	0	1	1.4	
Tx	0	0	3	4.1	
Nodal stage					
N0	19	29.7	24	32.9	0.973
N1	20	31.3	23	31.5	
N2	16	25.0	17	23.3	
N3	9	14.1	9	12.3	
TNM stage					
I	14	21.9	21	28.8	0.606
II	25	39.1	24	32.8	
III	25	39.1	28	38.4	
HR status					
ER− and PR−	24	37.5	13	17.8	0.01
ER+ and/or PR+	40	62.5	60	82.2	
HER2 status					
Positive	64	100	4	5.5	0.000
Negative	0	0	52	71.2	
Unknown	0	0	17	23.3	

Abbreviations: BMI=body mass index; HD=heart diseases identified in history; HTN=hypertension; DM=diabetes mellitus; HR=hormone receptor; ER=estrogen receptor; PR=progesterone receptor; HER2= human epidermal receptor 2.

TMN stage: 7^th^ American Joint Committee on Cancer (AJCC) TNM staging system for breast cancer

Systemic and locoregional treatments are detailed in Table 2. There was no significant difference in the frequency of anthracycline-based chemotherapy and cumulative dose of anthracycline between the concurrent-trastuzumab and no-trastuzumab cohort in both the neoadjuvant and adjuvant setting. Patients with HR positive disease were less likely to receive hormone therapy in the concurrent-trastuzumab cohort (*P* = 0.005). This might be due to more cases with HR positive of 1% to 9% in the concurrent-trastuzumab cohort (6 of 64 patients) compared with the no-trastuzumab cohort (0 of 73 patients) (*P* = 0.01). Internal mammary chain (IMC) RT was associated with significantly higher cardiac dose, the mean heart dose and mean dose to the LV was 1150.5 ± 230.9 cGy *versus* 568.9 ± 205.4 cGy(*P* = 0.000) and 1109.7 ± 397.9cGy *versus* 810.8 ± 276.1cGy(*P* = 0.013)respectively in patients with and without IMC RT. The proportion of IMC RT was lower in patients who received concurrent trastuzumab compared with those who did not receive trastuzumab (10.9% *versus* 31.5%, P = 0.004, Table 2).

**Table 2:** Details of systemic and locoregional treatment in 137 patients

Characteristics	Concurrent trastuzumab		No trastuzumab		*P* value
	N (64)	%	N (73)	%	
Chemotherapy					
Neoadjuvant only	1	1.6	0	0	0.032
Adjuvant only	52	81.3	59	80.8	
Both	11	17.2	7	9.6	
Neither	0	0	7	9.6	
Neoadjuvant chemotherapy					
Duration (months), median (range)	3 (0.6-4.4)		2.6 (0.1-3.5)		0.612
Neoadjuvant chemotherapy agent					
Taxanes/no anthracycline	10	83.3	5	71.4	0.097
Anthracycline/no taxanes	0	0	0	0	
Taxanes and anthracycline	0	0	2	28.6	
Others	2	16.7	0	0	
Adjuvant chemotherapy					
Duration (months), median (range)	3.7 (1.2-6.5)		4.2 (2-6.7)		0.372
Adjuvant chemotherapy agent					
Taxanes/no anthracycline	13	20.3	5	6.8	0.023
Anthracycline/no taxanes	7	10.9	10	13.7	
Taxanes and anthracycline	43	67.2	50	68.5	
Others	1	1.6	8	11.0	
Cumulative dose of anthracycline					
Total dose (mg), median (range)	445 (0-840)		450 (0-900)		0.513
Cumulative dose of taxanes					
Total dose (mg), median (range)	820 (0-2450)		595 (0-2480)		0.051
Start Trastuzumab					
Prior to RT	55	85.9	-	-	-
During RT	9	14.1	-	-	
Cumulative dose of trastuzumab before RT					
Total dose (mg), median (range)	1830 (0-4585)				
Cumulative dose of anthracycline					
Total dose (mg), median (range)	678 (192-1300)				
Hormone therapy in HR+ tumor					
TAM	23	57.5	38	63.3	0.015
AI	10	25	21	35	
None	7	17.5	1	16.7	
Surgery					
Mastectomy	41	64.1	40	54.8	0.271
Lumpectomy	23	35.9	33	45.2	
RT plan type					
FiF-IMRT	28	43.8	31	42.5	0.88
sIMRT	36	56.2	42	57.5	
IMC RT					
Yes	7	10.9	23	31.5	0.004
No	57	89.1	50	68.5	
RN RT					
SCV	37	57.8	20	27.4	
SCV+IMC	7	10.9	23	31.5	
None	20	31.3	30	41.4	

Abbreviations: RT=radiotherapy; TAM=tamoxifen; AI=aromatase inhibitor; FiF-IMRT=field-in-field forward-planned intensity-modulated radiotherapy; sIMRT=simplified multi-field inverse-planned IMRT; RN=regional nodes; IMC=internal mammary chain; SCV=supraclavicular lymph nodes.

Other abbreviations as in Table 1.

Trastuzumab was started, with neoadjuvant chemotherapy, with adjuvant chemotherapy, before initiation of RT and with RT in 10, 39, 6 and 9 patients, respectively. Trastuzumab regimen was consistent with chemotherapy schedule, which was administrated every 3 weeks and weekly in one and 9 patients during neoadjuvant chemotherapy, in 29 and 22 patients during adjuvant chemotherapy, respectively. All patients then received trastuzumab every 3 weeks during and after RT. There were four patients receiving concurrent administration of trastuzumab and anthracycline in the adjuvant setting. The median age of these four patients was 38.5 years (range 26-51). Sixty-three of the 64 patients completed 1-year trastuzumab as planned.

### Cardiotoxicity

Median follow-up of LVEF and clinical assessment of cardiac function from the initiation of RT was 6.7 months (range 3-60.9 months) and 26 months (range 6.4-60.9 months), respectively. In the concurrent-trastuzumab and non-trastuzumab cohort, the median absolute LVEF decrease from baseline to the lowest measured value after RT was 3% overall (range 7% increase to 15% decrease) and 1% overall (range 13% increase to 13% decrease), respectively. Grade 1 LVEF dysfunction (an asymptomatic decline in LVEF of at least 10% but less than 20% from baseline) occurred in 5 (7.8%) and 3 (4.1%) patients respectively. There was no significant difference in the rate of LVEF dysfunction between the concurrent-trastuzumab and no-trastuzumab cohort (7.8% *versus* 4.1%; *P* = 0.473). No patient presented chronic heart failure (CHF) or any cardiac symptoms whether they were or were not treated with trastuzumab. At the time of the last follow-up, LVEF of all patients recovered to normal (≥ 50%). In the concurrent-trastuzumab and non-trastuzumab cohort, the median time to recovery was 3.16 months and 3.33 months, respectively.

One patient stopped trastuzumab for developing pericardial effusion and chest pain after 15 cycles of 3-weekly scheme and 5 months after completion of RT. This is a 69-year-old woman with diabetes mellitus, but with no other cardiac risk factors. She had a T1N3 breast cancer with a baseline LVEF of 67% and received chest wall and Supraclavicular (SCV) irradiation of 50Gy. She was treated with 6 cycles of docetaxel and carboplatin adjuvant chemotherapy concurrently with trastuzumab. She did not developed LVEF dysfunction during the follow-up until 31 months from the initiation of RT. Her pericardial effusion was self-limited and recovered 1 month after stop of trastuzumab. The mean dose to the heart and left ventricle (LV) were 491.94cGy and 745.6cGy, respectively. The V_30_ of the heart and LV were 5% and 8%, respectively.

### Cardiac risk factors

Univariate analysis tested the effect of different patient- and treatment-related factors on the risk of LVEF dysfunction in patients treated with left-sided RT (Table 3). In the whole cohort, concurrent trastuzumab treatment was the only significant risk factor for absolute decrease of LVEF (*P* = 0.006), even if the rate of LVEF dysfunction did not differ significantly with regard to whether concurrent trastuzumab was given or not. Nor did the cumulative dose of anthracycline and IMC RT affect the rate of LVEF dysfunction. In the concurrent-trastuzumab cohort, IMC RT was of borderline significance (*P* = 0.088) in increasing the rate of LVEF dysfunction. Start trastuzumab during the period of RT instead of before RT increased the risk of LVEF dysfunction, although statistical significance was not found (22.2% *versus* 5.5%, *P* = 0.141). Young age (< 47 years) and pre/peri-menopause significantly increased the risk of LVEF absolute decrease. Neither cumulative dose of trastuzumab before nor during RT significantly influenced the risk of LVEF dysfunction.

**Table 3:** Univariate analysis of cardiac risk factors following left-sided RT

	Concurrent Trastuzumab						All patients					
Characteristics	LVEF dysfunction			LVEF decrease* (%)			LVEF dysfunction			LVEF decrease* (%)		
	N (64)	%	*P* value	Mean	SD	*P*-value	N (137)	%	*P*-value	Mean	SD	*P*-value
Overall	5	7.8		3% (−7% to 15%)			8	5.8		2% (−13% to 15%)		
Age (y)												
< 47	4/35	11.4	0.366	5	4.9	0.034	5/68	7.4	0.493	2.1	5.0	0.308
≥ 47	1/29	3.4		2	4.4		3/69	4.3		1.1	5.0	
BMI (kg/m^2^)												
< 23	4/39	10.3	0.64	5	4.9	0.127	5/80	6.3	1.0	1.8	4.9	0.646
≥ 23	1/25	4		2	4.6		3/57	5.3		1.3	5.2	
Comorbidity												
Yes	0/10	0	1	−0.5	3.5	0.053	0/23	0	0.356	1.1	4.3	0.615
No	5/54	9.3		3	4.9		8/114	7		1.7	5.1	
Menopausal status												
Pre/peri-menopausal	4/49	8.2	1	4	4.7	0.038	6/99	6.1	1.0	2.0	4.8	0.154
Post-menopausal	1/15	6.7		0	4.7		2/38	5.3		0.5	5.4	
Cumulative dose of anthracycline (mg)												
< 450	3/32	9.4	1	3	5.1	0.439	5/68	7.4	0.493	2.1	5.1	0.385
≥ 450	2/32	6.3		2	4.6		3/69	4.3		1.1	4.9	
Cumulative dose of taxanes (mg)												
< 720	2/28	7.1	1	3	5.1	0.731	3/68	4.4	0.718	1.1	5.5	0.588
≥ 720	3/36	8.3		2.5	4.7		5/69	7.2		1.9	4.5	
Hormone therapy												
Yes	4/40	10	0.642	3	4.6	0.531	5/92	5.4	0.718	1.1	4.8	0.097
No	1/24	4.2		3	5.2		3/45	6.7		2.5	5.3	
RT plan type												
FiF-IMRT	1/28	3.6	0.375	2.5	4.7	0.177	3/59	5.1	1.0	1.3	5.1	0.595
sIMRT	4/36	11.1		4	4.8		5/78	6.4		1.9	4.9	
IMC RT												
Yes	2/7	28.6	0.088	4	7.7	0.714	2/30	6.7	1.0	0.7	5.6	0.204
No	3/57	5.3		3	4.4		6/107	5.6		1.9	4.8	
Trastuzumab												
Yes	-	-	-	-	-	-	5/64	7.8	0.473	2.9	4.8	0.006
No	-	-		-	-		3/73	4.1		0.5	4.9	
Cumulative dose of trastuzumab before RT (mg)												
< 1830	2/32	6.3	1	3.5	4.7	0.497	-	-	-	-	-	-
≥ 1830	3/32	9.4		2.5	4.9		-	-		-	-	
Cumulative dose of trastuzumab during RT (mg)												
< 680	2/32	6.3	1	5	4.8	0.284	-	-	-	-	-	-
≥ 680	3/32	9.4		2.5	4.9		-	-		-	-	
Start of trastuzumab												
Before RT	3/55	5.5	0.141	2.5	4.6	0.191	-	-	-	-	-	-
During RT	2/9	22.2		5	5.5		-	-		-	-	

Abbreviations: SD=standard deviation;

Other abbreviations as in Table 1.

* Overall LVEF decrease expressed as median (range)

Multivariate analysis of risk factors for LVEF dysfunction was shown in Table 4. In 64 patients treated with concurrent trastuzumab, IMC RT and start trastuzumab during RT were found to be independent significant cardiac risk factors. In all 137 patients who received left-sided RT, concurrent trastuzumab treatment increased risk of LVEF dysfunction, but without statistical significance (*P* = 0.365).

**Table 4:** Multivariate analysis of LVEF dysfunction following left-sided RT

	Concurrent trastuzumab			All patients		
Characteristics	Hazard Ratio	95% CI	*P* value	Hazard Ratio	95% CI	*P* value
IMC RT						
Yes	1	-	0.016	1	-	0.6
No	0.038	0.003 - 0.542		0.618	0.102 - 3.733	
Start of trastuzumab						
Before RT	1	-	0.025	-	-	
During RT	20	1.469 - 272.319		-	-	
Trastuzumab						
Yes	-	-		1	-	0.365
No	-	-		0.504	0.114 - 2.219	

Abbreviations: LVEF=left ventricular ejection fraction; CI=confidence interval;

Other abbreviations as in Table 1.

### Dosimetric analysis in patients with concurrent treatment

**Figure 1:**
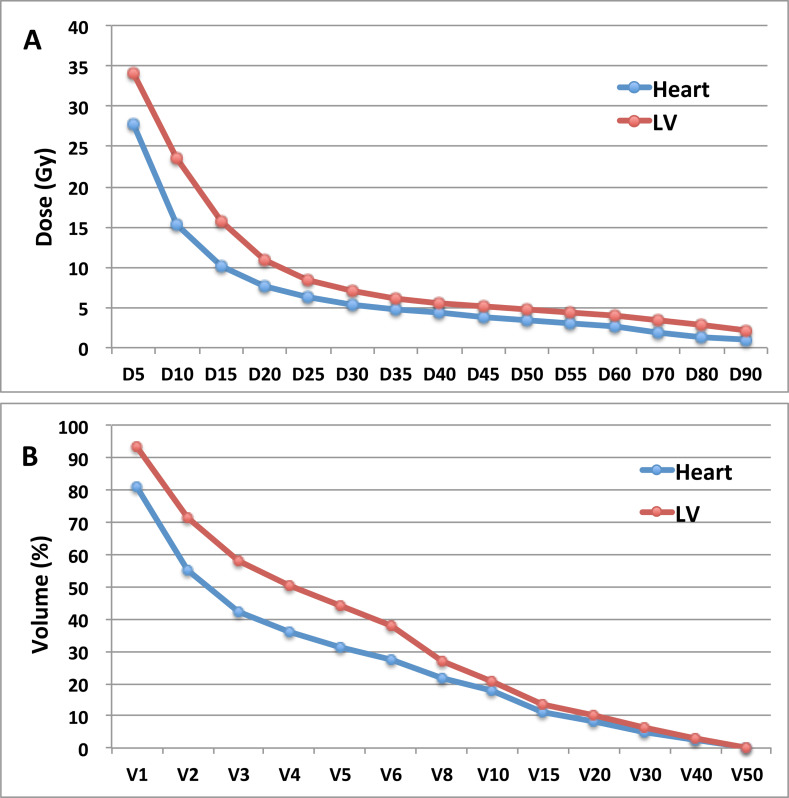
The average value of DVH parameters in 137 patients receiving left-sided RT **A.**, average Dn (Gy) of heart and LV; **B.**, average Vn (%) of heart and LV. DVH=dose volumes histograms; RT=radiotherapy; Dn=minimum dose which reached ≥n percentage of volume; LV= left ventricle; Vn=percentage of volume receiving ≥n Gy.

The average value of Dose-volume histogram (DVH) parameters of the heart and LV was summarized in Figure 1. The mean doses to the heart and LV were 633.5 ± 276.4c Gy and 843.9 ± 303.1cGy, respectively.

**Figure 2:**
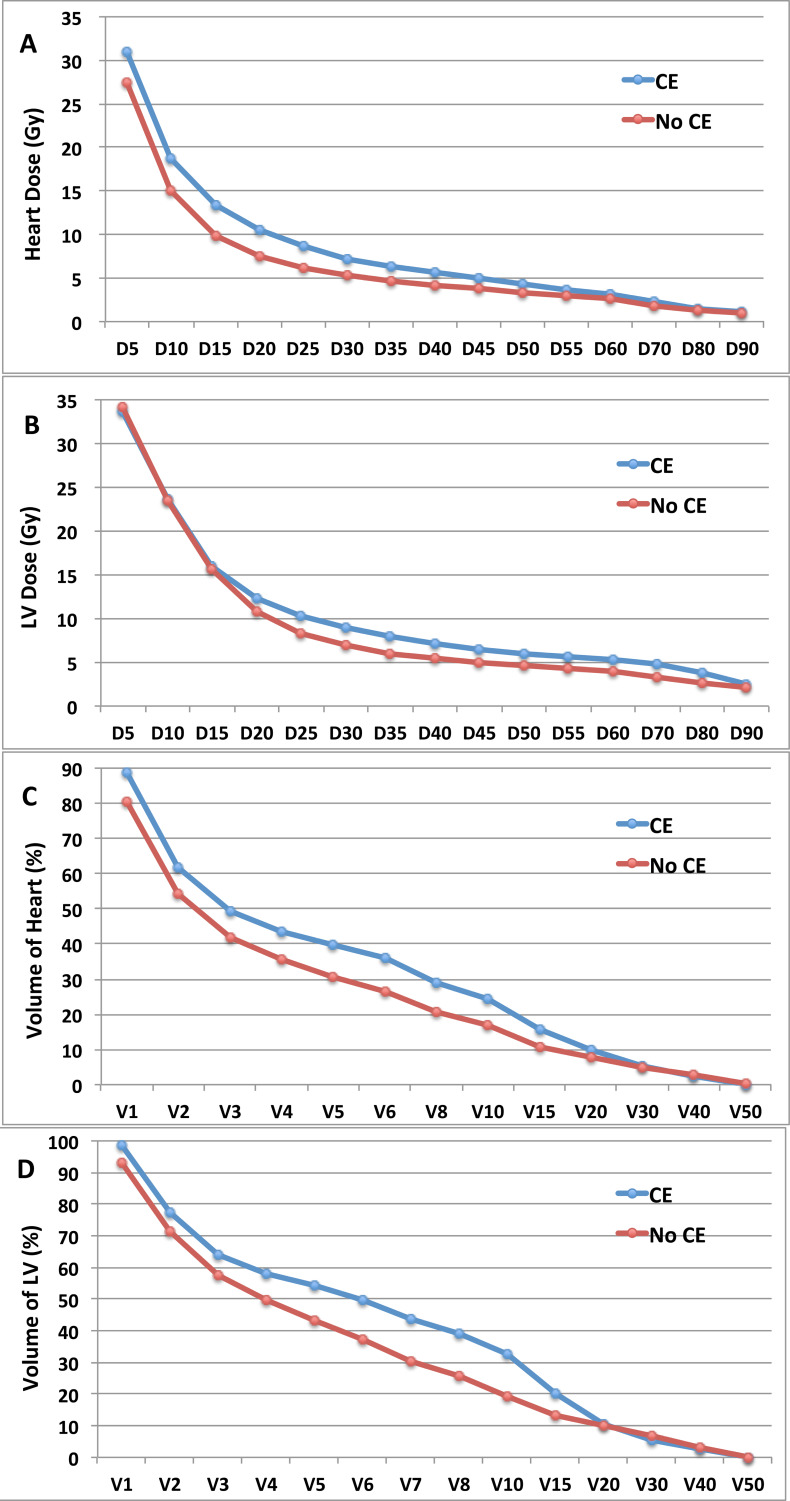
The average value of DVH parameters in 64 patients treated with concurrent trastuzumab, according to development of LVEF dysfunction **A.**, average value of Dn (Gy) of heart; **B.**, average value of Dn (Gy) of LV; **C.**, average value of Vn (%) of heart, **D.** average value of Vn (%) of LV. DVH=dose volumes histograms; CE=cardiac events; LVEF= left ventricular ejection fraction; Dn=minimum dose which reached ≥n percentage of volume; LV= left ventricle; Vn=percentage of volume receiving ≥n Gy.

The average mean dose delivered to the heart was significantly higher in those developing LVEF dysfunction compared to those with no observed cardiac toxicity (936.1± 333.7cGy *versus* 607.5 ± 258.3cGy; *P* = 0.01). Further analysis to compare the average value of dose-volume parameters of the heart and LV between patients with and without LVEF dysfunction found that, for the heart, D_10_-D_30_, D_50_-D_55_ and V_5_ -V_20_ were significantly higher in patients who developed LVEF dysfunction (all *P* < 0.05, Figure 2A and 2C). As to the LV, a continuous increase of D_30_-D_45_, D_65_-D_75_ and V_6_-V_15_ was also statistically significant in patients with LVEF dysfunction (all *P* < 0.05, Figure 2B and 2D). In multiple logistic regression analysis of all above statistically significant dose-volume histogram parameters, V_10_ of the LV was showed to be the best predictive factor for LVEF dysfunction in the setting of concurrent treatment. The V_10_ of the LV was 33.4±17.3% and 16.3±12.7% in patients with and without LVEF dysfunction, respectively (*P* = 0.01).

## DISCUSSION

Our data demonstrated that in patients with normal baseline cardiac function after adjuvant chemotherapy, concurrent administration of trastuzumab and left-sided RT was well tolerated using modern techniques, with a 7.8% grade 1 LVEF dysfunction rate in the concurrent-trastuzumab cohort and a 4.1% rate in the no-trastuzumab cohort. We also further confirmed a direct relationship between the dose-volume of cardiac structures irradiated and the risk of early cardiac toxicity in patients treated with concurrent trastuzumab and left-sided RT, which has been shown in our previous study [23].

Within 7-month median follow-up of LVEF, only five out of 64 patients (7.8%) developed grade 1 LVEF dysfunction after concurrent trastuzumab and left-sided RT. Only one patient presented self-limited pericardial effusion 5 months after completion of RT and no one developed CHF. In a reanalysis of adverse events data from the N9831 trial, the reported cumulative incidence of CHF and cardiac death was between 1.7% and 2.7% in the trastuzumab group [24]. Within a median follow-up of 16 months, Belkacemi *et al.* [25] [26] found that 9 of 92 patients developed a grade ≥ 2 of LVEF decrease after concurrent trastuzumab with RT. Shaffer et al. [27] reported that 18.5% patients stopped trastuzumab due to cardiac events after concurrent left-sided RT during a median follow-up of 15 months. The frequency of cardiac toxicity in our study seems much lower than previous reports. As follow up time of our study is relatively shorter than prior studies, some of the cardiac injury may still remain in sub-clinical phase, which would underestimate the actual cardiac events. Intensity-modulated radiotherapy (IMRT) has shown promising results as a cardiac sparing technique for breast cancer patients in several reports [28]. All cases in our study were treated with IMRT, which allowed for better cardiac protection and might therefore reduce the risk of cardiotoxicity. Old age (≥ 60 years) has been reported to be associated with increased trastuzumab related-LVEF dysfunction [29]. Only 6 patients (9.3%) were 60 years old or above in our study, which might also contribute to the low cardiotoxicity incidence.

So far, published studies indicated that concurrent administration of RT and trastuzumab is safe for the heart. However, so far, no study has ever done a straight comparison between concurrent trastuzumab with left-sided RT and left-sided RT alone. Our data showed that concurrent trastuzumab treatment was the only significant risk factor for absolute LVEF decrease (mean 2.9±4.8% *versus* 0.5±4.9%, *P* = 0.006). The rate of LVEF dysfunction in patients who received concurrent trastuzumab was almost twice as high as in their counterparts (7.8% *versus* 4.1%), even though without significant difference. More patients in the non-trastuzumab cohort received IMC RT, which might partly offset the difference of cardiac toxicity risk. Comparable to our findings, in the N9831 trial, the rate of cardiac events was 2.2% in the trastuzumab group and 0.4% in the non-trastuzumab group after a median follow-up time of 3.7 years. But left-sided patients were not analyzed in isolation in that study. To date, concurrent treatment of trastuzumab and left-sided RT is reported to be well tolerated, nevertheless, our data cautioned that the impact of concurrent trastuzumab on the risk of RT-related cardiac toxicity might exist to some extent, which warrants further follow up.

Detailed information of radiation doses received by cardiac structures was considered to be of paramount importance for a better correlation to the cardiotoxicity of concurrent trastuzumab and RT. To our knowledge, a retrospective study reported by Shaffer et al [27] is the only study prior to our work exploring this issue, which found no significant correlation. However, in this study irradiation techniques and dose fractionation were heterogeneous and the DVH data of the heart were undetailed, which would limit the applicability of the conclusion. Recently, our group published a study, which for the first time established a direct association between dose-volume of cardiac structures and frequency of early cardiac toxicity in the setting of concurrent trastuzumab and left-sided RT. After that study, we continually enrolled new patients such that our finding could be strengthened and extrapolated. In a series of 64 left-sided patients receiving concurrent trastuzumab, the association between dose-volume of cardiac structures and early cardiac toxicity was confirmed, which might provide information for practical recommendations with regard to dose constraints in treatment planning. The mean heart dose was significantly higher in those who developed LVEF dysfunction (936.1 ± 333.7cGy *versus* 607.5 ± 258.3cG y, *P* = 0.01). Darby et al. [30] reported that increase of cardiac events was proportional to mean heart dose. In the era of comprehensive treatment, several systemic agents might also increase the risk of cardiac damage in the setting of concurrent trastuzumab and radiation therapy. A high cumulative dose and concurrent use of anthracycline were shown to increase the risk of trastuzumab-associated cardiotoxicity [31]. In recent years, the improvements in breast cancer RT have kept the irradiation exposure of the heart at a very low level [8]. Nevertheless, even in such context our data suggested that constraint of the dose-volume of cardiac structures irradiated would remain an effective protective strategy for the heart.

In this study, DVH parameters that significantly correlated with the risk of LVEF dysfunction referred mainly to the low-intermediate dose volumes of the heart, with D_10_-D_30_, D_50_-D_55_ and V_5_-V_20_ of the heart and D_30_-D_45_, D_65_-D_75_ and V_6_-V_15_ of the LV. The clinical significance of relatively low dose volumes of the heart was seldom reported and controversial. In 32 breast cancer patients treated with left-sided RT, Chung et al. [32] found no correlation for perfusion and very low doses (mean heart dose < 5 Gy and D_95_) delivered to cardiac structures. IMRT has been introduced in the breast cancer RT to improve dose homogeneity in the breast as well as to reduce cardiac dose [28]. Compared with three-dimensional conformal radiotherapy (3D-CRT), IMRT significantly changed the pattern of irradiated dose distribution to the heart with increased mean dose and low dose volumes and decrease in high dose volumes [34, 35]. Currently there is no consensus whether such increase in low-dose volume is detrimental for heart, although it has become the major pitfall of IMRT being accepted as standard practice in breast RT. Further to our previous study, V_10_ of the LV, which represented low dose-volume, was found to be a significant prognostic factor of LVEF dysfunction. The absolute value of V_10_ in patients with LVEF dysfunction was two times as high as in patients with normal LVEF (33.4±17.3% *versus* 16.3±12.7%). Even if it maybe not yet to define one single dosimetric value as threshold, information from our study, together with future data, will help to develop a practical algorithm of dose-volume recommendations in IMRT of the left breast in the background of modern multidisciplinary treatment.

Despite the fact that IMC was part of the regional nodes irradiated in randomized trials that have proved the survival benefit of post-mastectomy radiotherapy in node-positive breast cancer patients [36-40], as well as in the recent published EORTC 22922/10925 and MA 20 trials which have proved that regional nodes irradiation in addition to breast/chest wall irradiation significantly improved distant disease-free survival in node-positive and high-risk node negative patients [41, 42], the benefit of IMC RT as part of regional RT is still controversial. Whatever technique to choose, IMC RT, more or less, is associated with an extra exposure of cardiac structures. Acute cardiotoxicity of concurrent trastuzumab and RT including IMC has been explored in several studies, with controversial results. In a retrospective study, Shaffer *et al.* [27] found that cardiac events were even fewer in patients who received left-sided IMC RT. By no means that IMC RT would serve as a protective factor for heart. Such counter-intuitive result may be due to small sample and bias in the selection of IMC RT. In another prospective study, 88 out of 106 patients received IMC RT, including 40 patients with left-sided breast cancer [43]. After a median follow-up of 28 months, 5 patients developed ≥ grade 2 left ventricular systolic dysfunction. The frequency of cardiac events was much higher than our results, which might indirectly reflect the detrimental effect of IMC RT. Our current study for the first time proved that left-sided IMC RT, in concurrent with trastuzumab, was an independent risk factor for LVEF dysfunction. Therefore, cardiac volume sparing and strict selection of high risk IMC irradiation are highly recommended [25].

Another phenomenon we observed was that the sequence of trastuzumab and RT was an independent risk factor influencing the cardiac toxicity. Start trastuzumab during the period of RT was associated with a higher risk of LVEF dysfunction, compared with start trastuzumab before the initiation of RT. As the baseline LVEF was assessed before RT, those who started trastuzumab during RT were almost simultaneously exposed to double cardiotoxic therapeutic modalities, which was quite different from those who exposed to trastuzumab first and RT subsequently. This result suggested that trastuzumab and left-sided RT have at least combined effect on cardiac toxicity. Therefore, those trastuzumab naïve patients who were not assessed for potential tolerance of trastuzumab and, prepared to double treatments might require stricter dose-volume constraints in treatment planning.

The main limitation of current study is that LVEF is not sensitive enough to detect early and minor cardiac dysfunction, thus the actual toxicity might be underestimated. Our group has also explored the role of diastolic function in monitoring early cardiac abnormalities under the same clinical scenario [44]. Nevertheless, as a fundamental parameter of cardiac function, LVEF is less susceptible to intra-observer differences. Therefore, LVEF would remain as the golden standard for monitoring cardiac function under current therapeutic modality [14]. New diagnostic modalities such as myocardial deformation imaging or biomarkers might help detect cardiac toxicity at an earlier stage [45-47]. In addition, biomarker such as HER2 [Ile655Val] genetic polymorphism and troponin I might help identify patients at high risk of trastuzumab-related cardiotoxicity [48, 49]. Several agents have been reported to ameliorate anthracycline associated cardiac toxicity, such as composite polymer nanoparticle and dihydromyricetin [50, 51]. Whether there agents will ameliorate trastuzumab- or radiation-associated cardiotoxicity needs further exploration. The median follow-up period of 26 months in this study is still too short. Considering the very long latent period of RT-induced cardiac toxicity, as well as the unclear long-term impact of trastuzumab on cardiac function, patients should be followed-up regularly for as long as possible, so that the clinical impact of early LVEF decrease will be further validated.

## MATERIALS AND METHODS

### Patients

The medical records of patients treated with adjuvant RT for left-sided stage I-III (AJCC, 7^th^ [52]) breast cancer between February 2009 and September 2011 were retrospectively reviewed. Patients with HER2+ disease (immunohistochemistry score 3 or fluorescence *in situ* hybridization positive) and also node-positive or high-risk node-negative disease were eligible for treatment with trastuzumab. Normal LVEF (≥ 50%) before the initiation of trastuzumab and RT were required. Demographic data and potential cardiac risk factors were collected, as well as histopathological, systemic and locoregional treatment details. The research protocol was reviewed and approved by the Ethical Committee and Institutional Review Board of the Fudan University Shanghai Cancer Center (FDSCC). All patients in FDSCC provided written informed consent.

### Systemic treatment

Neoadjuvant chemotherapy was administrated in patients with a primary diagnosis of stage IIA-IIIA disease. Adjuvant chemotherapy was decided by the multidisciplinary breast cancer team. Hormonal therapy was given to patients with HR positive breast cancers as determined by immunohistochemistry (> 1% positive). Trastuzumab was administered every 3 weeks (6 mg/kg after a first cycle of 8 mg/kg) or every week (2 mg/kg after a first cycle of 4 mg/kg).

### Radiotherapy and dosimetric analysis

RT was delivered to the whole breast or chest wall (50 Gy/25 fractions/5 weeks). Tumor bed boost of 10 Gy in 5 fractions was given using electron for breast conserving patients. SCV and IMC lymph nodes irradiation were given at the discretion of the radiation oncologist. No scar boost was used in patients receiving chest wall RT. All patients underwent CT-simulation in a supine position, immobilized on a breast board (Med-Tec, Inc. Orange City, IA, USA) with both arms abducted and raised overhead. For chest wall RT, field-in-field forward-planned intensity-modulated radiotherapy (FiF-IMRT) was used [53]. Breast RT was performed with simplified multi-field inverse-planned IMRT (sIMRT), which was described in details in a previous publication [23]. For patients treated with sIMRT, irradiation of SCV and IMC lymph nodes was integrated into sIMRT plan. For patients treated with FiF-IMRT, an anterior mixed photon and electron beam was used.

Cardiac structures were delineated according to the heart atlas published by Feng *et al*.[54]. A radiation oncologist, under supervision of a radiologist, deliberately contoured the whole heart and LV. DVH parameters of the heart and LV were calculated using individual dose distribution data in the RT planning system (ADAC Pinnacle 3, versions 8.0m). DVH parameters of the heart and the LV were collected and included the minimum dose reaching relative volume levels ranging from 1% to 95% (D_1_ to D_95_) and the percentage of volume exceeding 1 to 50 Gy (V_1_ to V_50_). As contribution of the tumor bed boost and SCV irradiation to the heart dose was very low [55, 56], adjustments for cardiac dose were not carried out for these patients.

### Statistical analysis

Cardiac event was evaluated according to LVEF decrease from the baseline measure. The LVEF measured by echocardiogram and/or multiple gated acquisition scan (MUGA). This assessment, combined with the clinical assessment of cardiac function, was performed before initiation of RT and every 3 to 6 months after RT for the first 5 years and annually thereafter. Cardiac event (grade ≥ 1) assessed according to the National Cancer Institute Common Toxicity Criteria (NCI-CTC, version 2.0).

Comparisons between groups were assessed using the chi-squared test (or Fisher's exact test) for categorical variables and Mann-Whitney U test for quantitative variables. Comparison between groups for dosimetric details was analyzed using the Student's *t*-test. A stepwise backward procedure was used to construct a set of independent risk factors of cardiac events. All factors achieving a P value < 0.10 were considered and sequentially removed if the P value in the multiple models was > 0.05. All P values were two-sided, *P* < 0.05 was considered significant. SPSS software version 21.0 was used.
